# Comparative Codon Usage Bias of CD2AP and BACH2 Across 49 Vertebrates: Implications for Porcine Macrophage Immunity in *Mycoplasma hyopneumoniae* Infection

**DOI:** 10.3390/biology15050389

**Published:** 2026-02-27

**Authors:** Wenxi Li, Peihuan Wang, Jiaxin Liu, Xiaoshu Xue, Shuhao Fan, Yueyun Ding, Xiaodong Zhang, Zongjun Yin, Xianrui Zheng

**Affiliations:** College of Animal Science and Technology, Anhui Agricultural University, Hefei 230036, China; 15543601529@163.com (W.L.); peihuan76@gmail.com (P.W.); winkxin2003@163.com (J.L.); xuexiaoshu0812@126.com (X.X.); shuhaowudi@163.com (S.F.); dingyueyun@ahau.edu.cn (Y.D.); xdzhang1983@ahau.edu.cn (X.Z.)

**Keywords:** CD2AP, BACH2, codon usage bias, mutation pressure, natural selection, immune response

## Abstract

Alveolar macrophages orchestrate phagocytosis and inflammatory programs during respiratory infection. CD2-associated protein (CD2AP) and BTB and CNC homology 2 (BACH2) are immune-related genes involved in cytoskeletal organization/vesicular trafficking and transcriptional regulation, respectively, but the coding-level constraints shaping their synonymous-site architecture remain unclear. Here, we profiled codon usage bias (CUB) of CD2AP and BACH2 across 49 vertebrate species using nucleotide composition, relative synonymous codon usage, and complementary codon bias diagnostics. Across species, BACH2 preferentially used G/C-ending codons with higher GC3s, whereas CD2AP was enriched for A/T-ending codons with lower GC3s. Both genes showed weak-to-moderate CUB (high ENC and modest CAI). ENC–GC3s and PR2 analyses indicated a strong compositional background at third codon positions, while neutrality analysis yielded shallow GC12–GC3 slopes, suggesting overall coding constraints, with compositional effects acting as a background influence and selective constraints possibly contributing to GC1/GC2 patterns. CD2AP deviated more from composition-only expectations than BACH2, suggesting greater gene-specific modulation. Phylogenetic reconstruction placed *Sus scrofa* within mammalian clades for both genes. In conclusion, CD2AP and BACH2 display opposite third-base codon-ending preferences across vertebrates, with CD2AP favoring A/T-ending codons and BACH2 favoring G/C-ending codons. This provides a comparative baseline for codon usage analyses of macrophage-relevant immune genes.

## Simple Summary

Respiratory disease caused by *Mycoplasma hyopneumoniae* reduces pig welfare and farm productivity. Lung immune cells called alveolar macrophages help control this infection, and their response depends in part on how efficiently key immune genes are made into proteins. The genetic code uses three-letter “words” to build proteins, and different animals can prefer different words even when they produce the same protein building block. In this study, we compared these preferences for two immune-related genes, CD2-associated protein and BTB and CNC homology 2, across 49 vertebrate species. CD2-associated protein tended to use genetic words ending in adenine or thymine, whereas BTB and CNC homology 2 more often used words ending in guanine or cytosine. Although the two genes showed opposite preferences, both displayed a similar overall level of preference across species. These results show that evolution shapes how immune genes are written, which may influence how readily they are produced during infection. The findings provide a comparative reference for pig respiratory immunity research and support gene design for future functional studies.

## 1. Introduction

*Mycoplasma hyopneumoniae* (*M. hyopneumoniae*), a major respiratory pathogen affecting swine production worldwide, causes chronic bronchopneumonia and leads to respiratory distress, stunted growth, and reduced feed conversion efficiency, thereby compromising productivity and economic returns in finishing pigs [1,2]. Porcine alveolar macrophages (PAMs) are among the first immune cells responding to *M. hyopneumoniae* infection and play central roles in initiating and shaping host inflammatory responses [3,4]. PAMs recognize invading pathogens and initiate phagocytosis, while coordinating immune regulation through cytokine production and inflammatory signaling pathways [4]. In experimentally infected pigs, pro-inflammatory cytokines are induced during infection [5]. Mechanistic studies further suggest that *M. hyopneumoniae* can activate innate signaling (e.g., NOD1-related pathways) to modulate inflammation [6] and interfere with macrophage apoptosis and M2 polarization to facilitate persistence [7]. Transcriptional programs and transcription factors (TFs) also contribute to these infection responses by regulating downstream immune and inflammation-related genes [3]. Importantly, synonymous codon usage can influence gene expression through effects on translation efficiency, mRNA stability, and transcription-related processes [8,9,10,11,12]. However, systematic analyses of codon usage patterns for host immune genes induced in PAMs after *M. hyopneumoniae* infection, including immune-relevant transcriptional regulators, remain limited, constraining our understanding of the sequence-level determinants of host immune efficiency and evolutionary adaptation. Although our analyses are based on annotated coding sequences that are not macrophage-specific per se, CD2AP and BACH2 have documented roles in macrophage function and pulmonary immune homeostasis; thus, comparative codon usage analysis can still reveal synonymous-site constraints that may affect the translation efficiency and related regulatory features of these macrophage-relevant immune genes.

CD2-associated protein (CD2AP) participates in signaling and vesicular trafficking by linking membrane proteins to the actin cytoskeleton, and has been extensively studied in kidney disease and Alzheimer’s disease [13,14,15,16]. Beyond these contexts, CD2AP also regulates actin remodeling and phagocytic vesicle trafficking in macrophages, thereby influencing pathogen internalization and clearance during respiratory infections [17,18]. BACH2 is a key TF with established roles in immune regulation and stress-related responses [19,20,21,22]. In macrophages, BACH2 modulates inflammatory signaling and prevents excessive activation, thereby maintaining alveolar macrophage function and pulmonary homeostasis during respiratory challenges [23,24]. Given the pivotal role of alveolar macrophages in *M. hyopneumoniae* infection and respiratory immune defense [3,6,7,25], both CD2AP and BACH2 represent critical regulators of macrophage-mediated pathogen clearance and inflammatory balance. Therefore, analyzing their codon usage patterns across species provides sequence-level insights into evolutionary constraints that may affect their expression efficiency and host adaptation during infection-related immune responses.

Codons are triplets of nucleotides composed of four bases (A, T, C, G) in DNA; during transcription, thymine (T) is replaced by uracil (U) in RNA [26,27,28]. Out of the 64 codons, three are stop codons (TAA, TAG, TGA), which terminate translation, while the remaining 61 encode 20 amino acids. Except for methionine (Met) and tryptophan (Trp), each encoded by a single codon (ATG for Met, TGG for Trp), all other amino acids are encoded by 2 to 6 synonymous codons [26]. Codon usage bias (CUB) refers to the non-random use of synonymous codons within genes or genomes, and it can vary among genes and across species. This bias is linked to biological factors such as tRNA abundance, protein levels, GC content, gene expression, mutation rates, and the secondary structure of mRNAs [8,27,29,30]. CUB reflects a gene’s evolutionary path shaped by mutations and natural selection [27,31,32], influencing transcription, translation efficiency, and protein synthesis [9,11,28,33]. As such, CUB is a crucial molecular marker for studying gene regulation and evolutionary adaptation. Efficient expression of host immune-related genes is essential for defense against pathogen invasion during infection [34,35].

Substantial research on codon preference has focused on pathogen genomes, including bacteria, fungi, and viruses [34,36,37]. In contrast, codon usage features of host immune-related genes that are relevant to macrophage functions in respiratory infection contexts remain less systematically characterized. To help fill this gap, we performed a cross-species codon usage analysis of CD2AP and BACH2 using curated CDSs from 49 vertebrate species and interpret the results in the context of porcine alveolar macrophage biology and *M. hyopneumoniae*-associated respiratory disease. In our analyses, GC3s represents GC content at the third codon position, and RSCU reflects standardized synonymous codon usage frequency. We calculated GC3s and RSCU and used the effective number of codons (ENC; also referred to as Nc) and codon adaptation index (CAI) to quantify CUB. We further evaluated codon bias patterns (GC3s, RSCU, ENC, CAI) and assessed the relative contributions of compositional bias and selection using complementary diagnostics. These results provide a comparative reference for codon usage features of macrophage-relevant immune genes and support future functional studies investigating potential codon-mediated effects on gene regulation.

## 2. Materials and Methods

### 2.1. Data Collection and Screening

A stratified sampling strategy across aquatic and terrestrial vertebrate taxa was used to reduce sampling bias and improve the stability of cross-species comparisons. Representative species were selected at the order level where possible. Coding sequences (CDSs) of BACH2 and CD2AP were retrieved from NCBI GenBank (accessed 15 October 2025) [38]. Only species with both gene sequences available were retained. Sequences containing nonstandard bases (N), premature stop codons, or lengths not divisible by three were excluded. For species with multiple transcripts, the longest transcript with the most complete annotation was selected. All sequences were checked for intact open reading frames using ORFfinder (NCBI) [39]. In total, 49 species were included. Abbreviated species names were used in figures for visual clarity (Table S1).

### 2.2. Phylogenetic Analysis

Multiple sequence alignments of CDSs were generated using MAFFT v7 [40]. Phylogenetic trees were reconstructed in MEGA11 using the neighbor-joining (NJ) method with the p-distance model and 1000 bootstrap replicates to assess node support [41]. Trees were built separately for BACH2 and CD2AP. Tree visualization and hierarchical clustering were additionally performed using ChiPlot (accessed 15 October 2025) [42]. For visual clarity, the two Suidae species included in the dataset (*Sus scrofa* and *Phacochoerus africanus*) were highlighted in the phylogenetic trees and selected comparative plots.

### 2.3. Codon and Nucleotide Composition Analysis

Codon usage indices were calculated using CodonW v1.4.4 [43], including third-base nucleotide content (A3s, T3s, C3s, G3s), overall GC and GC3s, the effective number of codons (ENC) [44], and the codon adaptation index (CAI) [45]. Stop codons and the single-codon amino acids (Met and Trp) were excluded from RSCU-based analyses (including PCA and optimal codon identification) to avoid skewing codon bias estimates. All outputs were compiled in Excel (Tables S2 and S3). CAI was computed in CodonW v1.4.4 using the built-in reference codon usage table (default setting) following Sharp and Li [45]. Because CAI is reference-dependent, we applied the same CAI setting to all species to keep the calculation framework consistent; therefore, CAI is interpreted primarily in a relative sense in cross-species comparisons.

### 2.4. Analysis of Relative Synonymous Codon Usage (RSCU)

RSCU was used to evaluate codon preference by comparing the observed usage of each synonymous codon with the expected usage under equal synonymous choice [27,46]. Stop codons were excluded from RSCU calculations. Codons with RSCU > 1 were considered over-represented (preferred), whereas codons with RSCU < 1 were considered under-represented. RSCU was computed as follows:

$$RSCU=\frac{X_{ij}}{\frac{1}{N_{i}}\sum_{j=1}^{N_{i}\;}{\;\;X}_{ij}}$$

where $X_{ij}$ is the observed count of the *j*-th codon for the *i*-th amino acid, and $N_{i}$ is the number of synonymous codons encoding that amino acid [46].

### 2.5. Optimal Codon Analysis

To identify optimal codons, species were stratified into a high-bias group (five species with the lowest ENC values) and a low-bias group (five species with the highest ENC values). For each codon, the difference in mean RSCU between groups was calculated as follows [43]:

$$\Delta {\mathrm{RSCU}}_{i}={\bar{\mathrm{RSCU}}}_{i,\mathrm{High}}-{\bar{\mathrm{RSCU}}}_{i,\mathrm{Low}}$$

Optimal codons were identified using the ΔRSCU method (ΔRSCU > 0.08) together with an RSCU contrast criterion (RSCU > 1 in the high-bias group and RSCU < 1 in the low-bias group), following previous studies [47,48].

### 2.6. Principal Component Analysis (PCA)

PCA was performed in R (v4.3.2) using ggplot2, factoextra, and ggrepel on a 59 × 49 RSCU matrix (59 synonymous codons; 49 species) [49,50,51,52]. Stop codons and the single-codon amino acids (Met and Trp) were excluded, yielding 59 synonymous codons for PCA. Species scores were used to visualize clustering patterns, and codon loadings were used to interpret the major axes. For codon contributions, a composite loading distance was calculated as follows:

$$d=\sqrt{{\left({\mathrm{loading}}_{\mathrm{PC}1}\right)}^{2}+{\left({\mathrm{loading}}_{\mathrm{PC}2}\right)}^{2}}$$

Codons with larger d values were highlighted in the loading plots.

### 2.7. Correlation Analysis

Pearson’s correlation coefficients (r) were computed among codon bias and nucleotide composition variables (e.g., A3s, T3s, C3s, G3s, ENC, CAI, GC, GC3s). Correlation heatmaps were generated using ggplot2 and corrplot [51,53].

### 2.8. ENC–CAI Analysis

ENC and CAI were analyzed by linear regression in GraphPad Prism 10 to assess the relationship between codon bias and codon adaptation. Pearson’s correlation coefficient was calculated as an overall measure of association [36,37,43,44,54].

### 2.9. PR2 Analysis

Parity Rule 2 (PR2) analysis was used to evaluate third-base nucleotide asymmetry and infer the relative roles of mutation pressure and selection in shaping synonymous codon usage [32,55].

### 2.10. ENC–GC3s Plot Analysis

ENC values were plotted against GC3s to assess whether codon usage patterns conform to the mutation-driven expectation. The expected ENC values were calculated as follows [44]:

$${\mathrm{ENC}}_{\exp}=2+S+\frac{29}{S^{2}+{\left(1-S\right)}^{2}}$$

where *S* = GC3s.

### 2.11. Neutrality Analysis

Neutrality analysis was performed by regressing GC12 (mean of GC1 and GC2) against GC3 to evaluate the relative contributions of mutation pressure and selection, where slopes approaching 1 suggest mutation dominance and slopes near 0 suggest stronger selective constraints [55].

### 2.12. K-Means Cluster Analysis

K-means clustering (k = 3, nstart = 100) was performed using standardized codon adaptation index (CAI) values of BACH2 and CD2AP to classify species into codon adaptation clusters and to visualize separation patterns [56]. Because CAI is reference-dependent, clustering was intended to summarize relative CAI patterns under the same CAI computation setting rather than to imply strict absolute comparability of CAI values across species.

## 3. Results

### 3.1. Nucleotide Composition Attributes of the CD2AP Gene and BACH2 TF Coding Sequence

**Figure 1:**
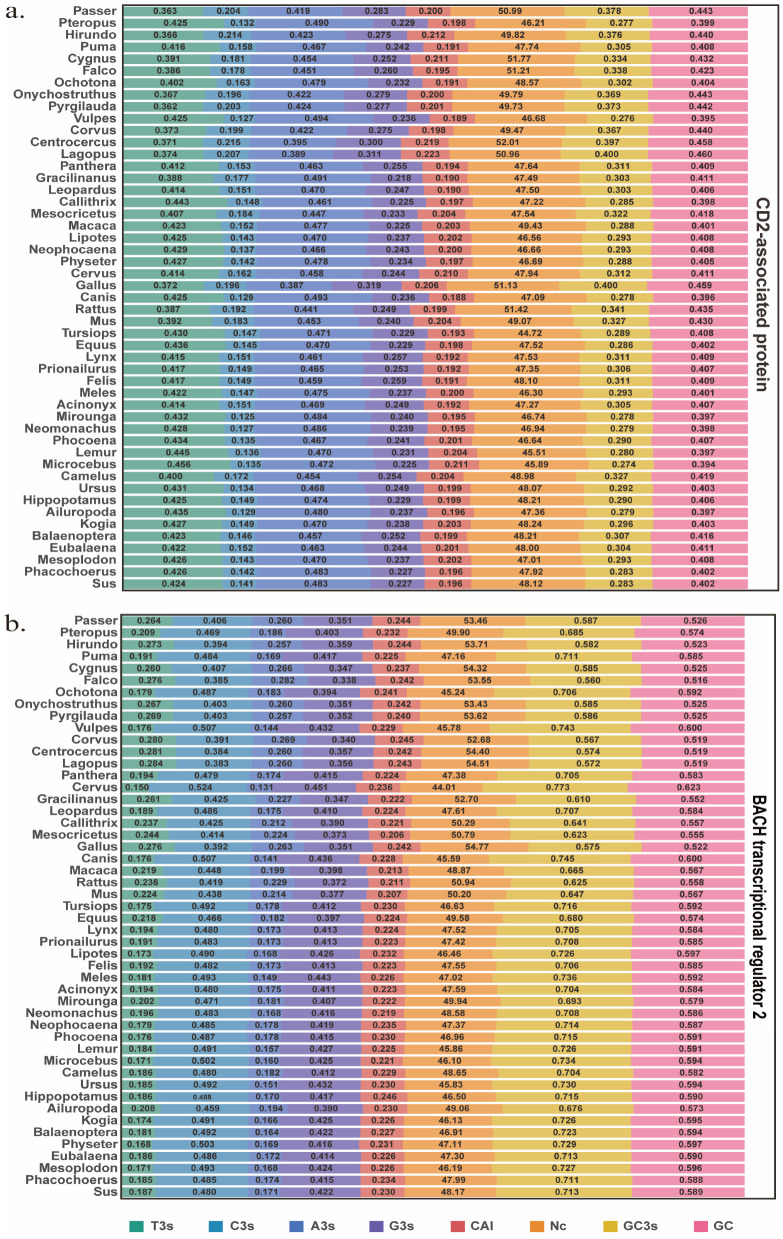
Comparative nucleotide composition and codon usage indices of CD2AP and BACH2 across 49 representative species. (**a**) Heatmap of third-codon-position nucleotide frequencies (T3s, C3s, A3s, G3s) and codon usage indices for CD2AP. (**b**) Corresponding results for BACH2 across the same species set. Values shown in each cell are the species-level estimates for codon adaptation index (CAI), effective number of codons (ENC; also referred to as Nc), GC content at the third codon position (GC3s), and overall GC content (GC).

We summarized CodonW-derived nucleotide composition and codon usage indices for CD2AP and BACH2 across 49 species (Figure 1a,b). CD2AP showed an AT-leaning third-position profile, with higher third-codon-position nucleotide proportions (mean ± SD) A3s and T3s than G3s and C3s (T3s 0.4115 ± 0.0240; C3s 0.1588 ± 0.0257; A3s 0.4599 ± 0.0259; G3s 0.2478 ± 0.0222). Consistent with this, CD2AP exhibited lower GC-related indices (GC3s 0.3121 ± 0.0358; GC 0.4140 ± 0.0180). In contrast, BACH2 displayed a GC-leaning third-position composition dominated by C3s and G3s (T3s 0.2093 ± 0.0386; C3s 0.4611 ± 0.0408; A3s 0.1942 ± 0.0405; G3s 0.3988 ± 0.0309), accompanied by higher GC3s and GC (GC3s 0.6775 ± 0.0601; GC 0.5719 ± 0.0285). Both genes showed modest codon adaptation (CAI 0.1994 ± 0.0074 for CD2AP; 0.2292 ± 0.0097 for BACH2) and weak-to-moderate overall codon bias, as reflected by ENC (48.14 ± 1.71 vs. 48.97 ± 2.99). Overall, the heatmap highlights a clear gene-dependent compositional tendency at synonymous third sites (CD2AP AT-leaning vs. BACH2 GC-leaning), while bias strength remains broadly comparable across species. Notably, *Sus scrofa* showed values within the range observed for other cetartiodactyl and mammalian species across the reported indices.

### 3.2. CD2AP Gene and BACH2 TF Codon Usage Patterns and Optimal Codons

**Figure 2:**
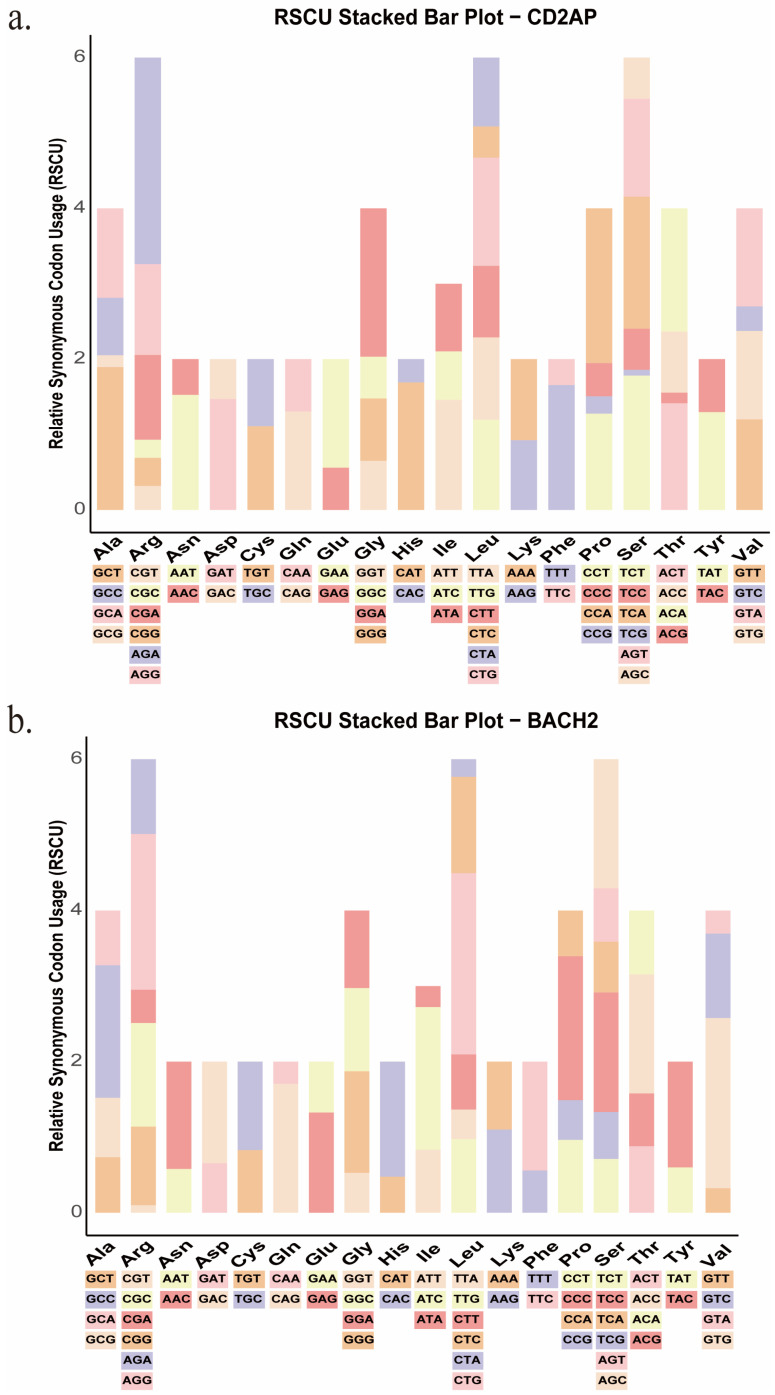
(**a**) CD2AP. (**b**) BACH2. Mean RSCU values were calculated from coding sequences of 49 species. The *x*-axis shows amino acids, with the corresponding synonymous codons listed below each bar; the *y*-axis indicates RSCU values. RSCU > 1 indicates preferential usage of a codon, whereas RSCU < 1 indicates under-representation. Colored segments represent individual synonymous codons as indicated beneath each amino acid group (stop codons excluded).

RSCU analysis revealed distinct synonymous codon usage preferences between CD2AP and BACH2 across 49 species (Figure 2a,b). In CD2AP, codons with RSCU > 1 were predominantly A/T-ending, accounting for 24 of 29 favored codons (82.8%), whereas G-ending codons were rare (5/29) and C-ending codons were absent. In contrast, BACH2 showed a strong enrichment of C/G-ending codons: Among the 25 favored codons (RSCU > 1), C-ending codons comprised 16/25 (64%) and G-ending codons comprised 8/25 (32%), with only one A-ending codon and no T-ending codons. These opposite third-base preferences are consistent with the compositional background of the two genes, with CD2AP exhibiting a lower GC3s tendency and BACH2 showing a higher GC3s tendency. Notably, five favored codons (CTG, GTG, AGG, GGA, and CAG) were shared between CD2AP and BACH2, suggesting conserved codon preferences for a subset of amino acids across species. Species-level clustering based on RSCU values is shown in the Supplementary Materials (Figure S1), supporting an overall lineage-related codon usage background with gene-specific deviations, consistent with established frameworks for codon bias evolution and its determinants [28,31,57]. To identify optimal codons, coding sequences were stratified into high- and low-codon bias groups and analyzed using CodonW v1.4.4. CD2AP contained four optimal codons (AGG, TGC, AGC, and ATC; Table S4), whereas BACH2 contained six (AGA, TTG, AAA, CCT, GCT, and ACT; Table S5). The complete lists of favored codons (RSCU > 1) and optimal codons are presented in the Supplementary Tables.

### 3.3. Multivariate Patterns and Clustering of Codon Usage Among Species

**Figure 3:**
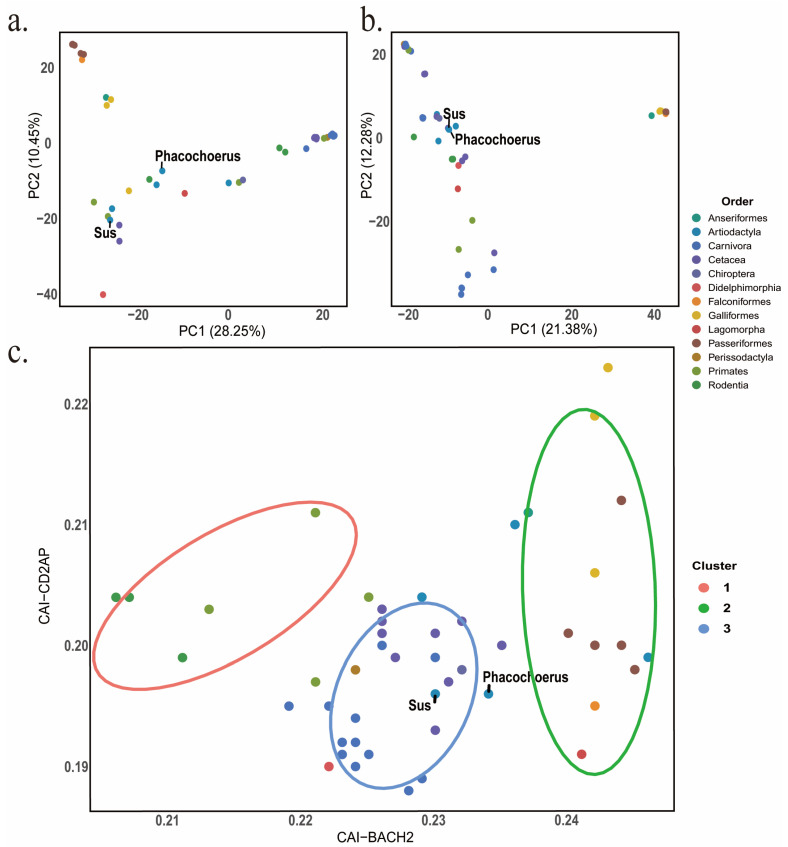
(**a**,**b**) PCA of RSCU profiles (59 synonymous codons) for CD2AP (**a**) and BACH2 (**b**) across 49 species; points are colored by taxonomic order, and the two Suidae species (*Sus scrofa* and *Phacochoerus africanus*) are labeled. Percent variance explained by PC1 and PC2 is shown on the axes. (**c**) K-means clustering (k = 3) of species in CAI space (CAI_BACH2 vs. CAI_CD2AP); points are colored by cluster, and *Sus scrofa* and *Phacochoerus africanus* are labeled.

We applied PCA to the RSCU dataset (59 synonymous codons; 49 species) to characterize cross-species variation in codon usage for CD2AP and BACH2 (Figure 3a,b). In CD2AP, PC1 and PC2 accounted for 28.25% and 10.45% of the total variance, respectively, whereas in BACH2 they explained 21.38% and 12.28%. In both PCA plots, species were broadly grouped by taxonomic order, with avian taxa tending to separate from most mammals primarily along PC1. The two Suidae species included in our dataset (*Sus scrofa* and *Phacochoerus africanus*) clustered with other mammals in both analyses, indicating that their RSCU profiles overlap with the Cetartiodactyla pattern observed in this dataset. To assess codon adaptation across the two genes, we projected species into a two-dimensional CAI space (CAI_BACH2 versus CAI_CD2AP) and performed k-means clustering with k = 3 (Figure 3c). This partitioned the 49 species into three clusters, indicating coordinated variation in CAI between CD2AP and BACH2. Both *Sus scrofa* and *Phacochoerus africanus* were located in the intermediate cluster, consistent with moderate codon adaptation for both genes compared with the remaining species. Codon-level loading patterns are shown in Figure S2, and correlations among codon usage indices are presented in Figure S3.

### 3.4. Factors Shaping Codon Usage Bias in CD2AP and BACH2

**Figure 4:**
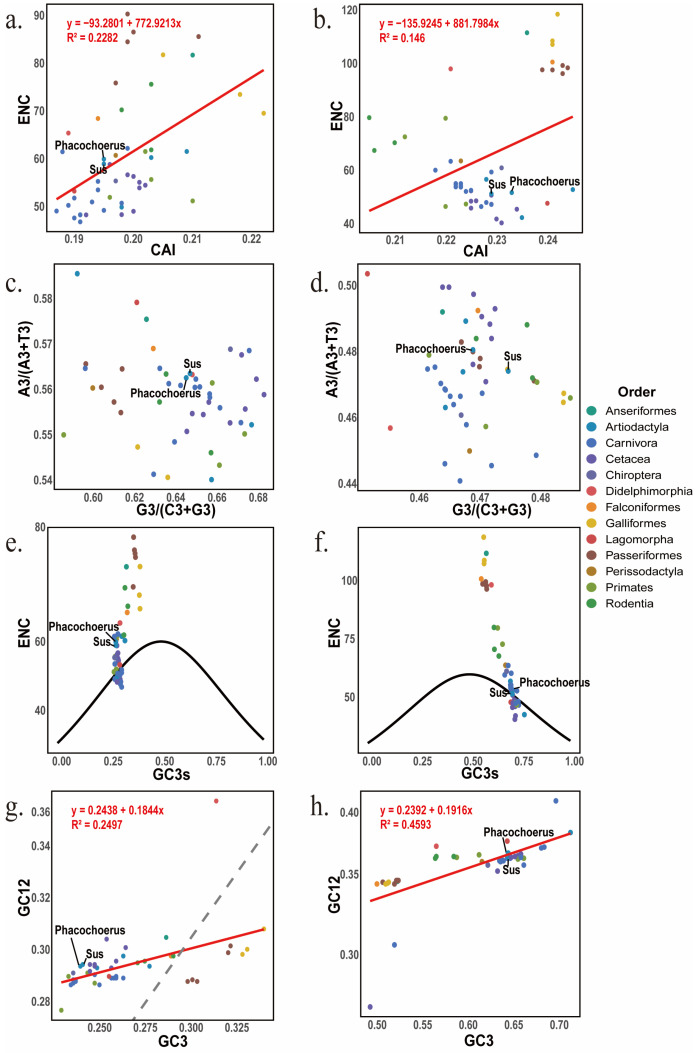
Factors shaping codon usage bias in CD2AP and BACH2 across 49 species. (**a**,**b**) ENC–CAI plots. (**c**,**d**) PR2 plots [A3/(A3 + T3) vs. G3/(G3 + C3)]. (**e**,**f**) ENC–GC3s plots with Wright’s expected curve (black). (**g**,**h**) Neutrality plots (GC12 vs. GC3) with linear regression (red). The dashed line indicates the y = x reference line (GC12 = GC3). Left panels: CD2AP; right panels: BACH2. Points are colored by taxonomic order, and the two Suidae species (*Sus scrofa* and *Phacochoerus africanus*) are labeled.

ENC–CAI patterns suggest that expression-related optimization (as approximated by CAI) is not the primary driver of overall codon bias (ENC) in either gene (Figure 4a,b). In both CD2AP and BACH2, ENC shows only a weak association with CAI (R^2^ = 0.2282 and 0.146), and the two Suidae species in our dataset (*Sus scrofa* and *Phacochoerus africanus*) fall within the broader order-level distribution, indicating a limited contribution of translational optimization to interspecies variation in CUB. PR2 plots further reveal gene-specific third-position nucleotide asymmetry (Figure 4c,d): CD2AP points are mainly distributed above 0.5 on both axes, consistent with a purine enrichment (A/G bias), whereas BACH2 tends to fall below 0.5, indicating a pyrimidine enrichment (C/T bias). Together, these patterns support compositional asymmetry as a major baseline shaping synonymous-site usage, with clear gene-dependent directionality.

ENC–GC3s analyses show that codon usage in both genes is closely linked to GC3s (Figure 4e,f), with CD2AP concentrated at low GC3s and BACH2 at high GC3s. While many species follow Wright’s expected curve, a subset deviates from the composition-only expectation, suggesting that compositional bias provides the background framework but does not fully account for codon usage variation across lineages. Consistently, neutrality plots reveal shallow GC12–GC3 regression slopes (≈0.1844 for CD2AP; ≈0.1916 for BACH2; Figure 4g,h), indicating limited propagation of third-position GC variation into first/second positions. These results are consistent with overall coding constraints and suggest that compositional/mutational effects at synonymous third sites act as a background influence, while selective constraints may play a relatively stronger role in shaping GC1/GC2 across taxa. Because the slope differences between the two genes are modest, we interpret the contrast between mutation- and selection-related patterns cautiously. Notably, BACH2 shows a slightly tighter GC12–GC3 association (higher R^2^), suggesting somewhat stronger coupling to compositional background compared with CD2AP.

### 3.5. Phylogenetic Tree Analysis of CD2AP and BACH2 Coding Sequences

**Figure 5:**
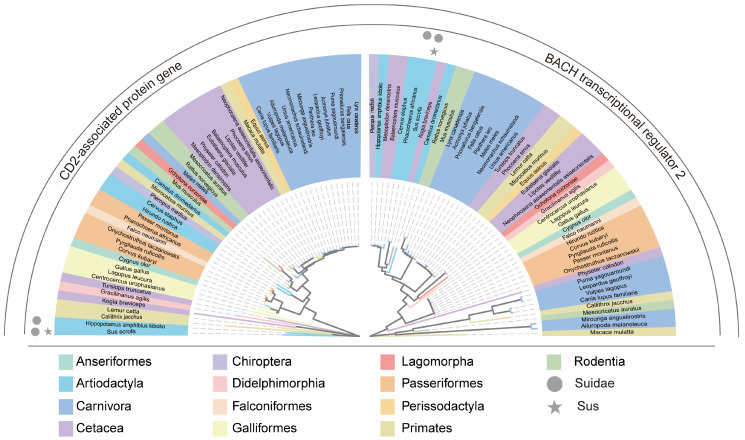
Phylogenetic trees of CD2AP and BACH2 across 49 species. NJ trees (MEGA11; 1000 bootstraps) based on coding sequences. Colors denote orders; the two Suidae species (*Sus scrofa* and *Phacochoerus africanus*) are highlighted (e.g., *Sus scrofa* by an asterisk and *Phacochoerus africanus* by a filled circle).

To place the codon usage results in an evolutionary context, we reconstructed phylogenetic trees for CD2AP and BACH2 using coding sequences from 49 species (Figure 5). For both genes, species largely grouped into well-supported clades consistent with established taxonomy at the class and order levels. The two Suidae species in our dataset (*Sus scrofa* and *Phacochoerus africanus*) clustered within Cetartiodactyla together with other cetartiodactyl species, matching their expected positions. The two trees showed broadly similar higher-level topologies, whereas minor differences were mainly observed near terminal branches, which is common for single-gene phylogenies. Overall, the order-level clustering in the phylogenies broadly aligns with our codon bias analyses, suggesting that shared evolutionary background contributes to interspecies variation, with gene-specific effects superimposed.

## 4. Discussion

In respiratory infections, alveolar macrophages act as frontline immune cells that coordinate phagocytosis and inflammatory signaling to shape host defense outcomes [3,4]. In swine, *M. hyopneumoniae* infection is associated with cytokine induction and immune dysregulation, underscoring the contribution of macrophage-mediated responses to disease progression [5]. CD2-associated protein (CD2AP) links membrane-associated signaling to actin remodeling and vesicular trafficking, supporting immune-cell structural dynamics and intracellular transport [15,16]. BACH2 is a transcription factor that contributes to immune regulation and maintenance of homeostasis during inflammatory or stress-related challenges [19,20]. Because synonymous codon usage can affect gene expression through translation efficiency, mRNA stability, and transcription-associated processes [12,28], clarifying codon usage features of macrophage-relevant host immune genes under infection-associated contexts may provide sequence-level insights into evolutionary constraints. Accordingly, we compared codon usage in CD2AP and BACH2 across 49 species [9,31].

Across species, CD2AP and BACH2 showed contrasting synonymous codon preferences. BACH2 displayed an enrichment of G/C-ending codons and higher GC3s, whereas CD2AP favored A/T-ending codons and lower GC3s, indicating distinct compositional tendencies at synonymous third positions. This divergence is most plausibly driven by gene-dependent compositional background and locus-specific mutational biases that shape GC3s, rather than by a universal expression-driven optimization. In vertebrate genomes, local genomic context can impose distinct GC baselines among loci, which strongly influence synonymous third positions. Beyond mutation/composition, additional gene-specific selective constraints may further modulate codon choice through effects on mRNA secondary structure/stability and translation kinetics. Although codon usage bias does not directly reflect protein function, different regulatory regimes and expression contexts may favor different synonymous-site architectures across genes [8,9,28]. Given that codon bias may encode regulatory information beyond amino acid sequence [8], these opposing patterns may reflect gene-specific evolutionary constraints aligned with their functional roles in macrophage biology. BACH2 participates in shaping immune programs and restraining excessive inflammatory activation [19,20,23,24], whereas CD2AP contributes to actin remodeling and vesicle trafficking relevant to macrophage internalization and cellular organization [15,16,17,18]. These results suggest that synonymous-site architectures can diverge even among macrophage-relevant loci, potentially reflecting distinct regulatory regimes.

Recent work has emphasized that codon usage patterns can reflect a balance between mutational/compositional biases and selective forces related to gene regulation and host adaptation, and that these effects can differ among loci and biological contexts. In line with these observations, our gene-dependent GC- versus AT-ending preferences suggest that CD2AP and BACH2 are shaped by distinct synonymous-site constraints across vertebrates. Moreover, studies in veterinary/pathogen systems have highlighted that codon usage analyses can help contextualize evolutionary pressures and host adaptation, motivating the integration of comparative codon usage with functional and infection-stage data. Therefore, our results provide a cross-species framework that can be extended by incorporating expression profiles and pathogen codon features, consistent with recent codon usage studies in animal pathogens [58,59].

Multiple diagnostics (ENC–CAI, ENC–GC3s, PR2, and neutrality analyses) indicate that synonymous codon usage in both genes is influenced by compositional bias at third positions, overlaid by broader selective constraints. For BACH2, codon usage variation closely tracks GC3s and generally conforms to the expected ENC–GC3s relationship, supporting a strong compositional baseline; PR2 asymmetry further indicates gene-specific nucleotide skews at synonymous sites [26,30]. However, neutrality plots show shallow GC12–GC3 slopes, suggesting limited propagation of GC3 variation into first/second positions and consistent with overall coding constraints across taxa [55]. CD2AP exhibits clearer departures from composition-only expectations (reduced ENC at comparable GC3s and greater dispersion around Wright’s curve) [44]. Together with PR2 patterns and the weak ENC–CAI association, these findings imply that additional selective or functional constraints likely contribute to shaping CD2AP synonymous-site usage across lineages [57]. Overall, compositional bias provides the background, while coding evolution appears to be subject to broader constraints, with selection potentially contributing alongside compositional effects.

Species-level multivariate analyses further contextualize these patterns. In PCA of RSCU values, *Sus scrofa* clusters with other mammals for both genes and overlaps with the Cetartiodactyla distribution, indicating that porcine codon usage is consistent with the broader Cetartiodactyla pattern observed in this dataset. K-means clustering in CAI space places pig in an intermediate cluster, consistent with moderate codon adaptation relative to other taxa. These observations support the view that codon usage at these loci is shaped primarily by lineage-related compositional context, with gene-specific deviations superimposed. Although host–pathogen codon usage similarity has been proposed to influence infection efficiency through host-side optimization or pathogen-side codon mimicry [32,35], direct evidence in swine–*Mycoplasma* systems remains limited. In this light, the mammal-typical codon usage profiles of porcine CD2AP and BACH2, together with gene-specific departures attributable to compositional and selective influences, are compatible with a broader coevolutionary framework. Future work integrating pathogen codon usage and infection-stage expression data will be needed to test whether host–pathogen codon usage compatibility contributes to immune efficiency and disease outcome [1,3,6,37].

This study provides a comparative reference for the codon usage features of two macrophage-relevant genes and a framework for codon-aware comparative immunogenomics [8,26]. Several limitations should be noted. First, our analyses relied on reference CDS annotations, and isoform choice or annotation quality may introduce noise [60]. Second, CAI is a reference-dependent metric; therefore, absolute CAI values should be interpreted cautiously in cross-species contexts. In this study, CAI-based analyses are intended primarily to summarize relative patterns under a unified computational setting. Third, although ENC, CAI, and related indices are informative, disentangling mutation pressure from selection remains challenging; thus, inferences regarding evolutionary forces should be interpreted probabilistically [44,55]. Fourth, we did not incorporate infection-condition expression profiles, tRNA abundance, or translatome evidence, which are needed to directly test translational selection and codon optimality during immune activation [8,9,35]. Finally, while host–pathogen codon usage coupling has been documented in viral systems [34,61,62], determining whether analogous mechanisms operate in the swine–*Mycoplasma* interaction requires integrative functional validation. Future studies combining infection-stage transcriptomics, tRNA profiling, and macrophage-based assays will be crucial for linking codon architecture to immune gene regulation, pathogen adaptation, and disease outcomes [3,7,8].

## 5. Conclusions

We characterized synonymous codon usage in CD2AP and BACH2 using coding sequences from 49 vertebrate species. The two genes displayed contrasting third-position composition and codon preferences: CD2AP was AT-leaning with enriched A/T-ending preferred codons, whereas BACH2 was GC-leaning with enriched C/G-ending preferred codons. Across taxa, codon bias diagnostics supported a prominent compositional baseline at synonymous third sites. Neutrality analysis yielded shallow GC12–GC3 slopes, consistent with overall coding constraints and suggesting limited propagation of GC3 variation into GC1/GC2. Compared with BACH2, CD2AP showed greater departures from composition-only expectations, consistent with more pronounced gene-specific modulation. In multivariate analyses and phylogenetic reconstruction, *Sus scrofa* followed the mammalian lineage background and fell within the range observed across Cetartiodactyla in our dataset. Collectively, these results provide a cross-species reference for codon usage features of two macrophage-relevant immune genes and motivate future work integrating infection-stage expression and pathogen codon profiles to test potential functional consequences of synonymous-site architecture.

## Data Availability

Data are contained within the article and Supplementary Materials.

## Supplementary Materials

The following supporting information can be downloaded at: https://www.mdpi.com/article/10.3390/biology15050389/s1, Figure S1. RSCU heatmaps and hierarchical clustering across species. Figure S2. Codon-level PCA loading plots based on RSCU. Figure S3. Correlation matrices of codon-usage indices. Table S1. Species List with Abbreviated Names and Corresponding CDS Accessions. Table S2. Codon Composition Metrics for CD2AP Gene. Table S3. Codon Composition Metrics for BACH2 Transcription Factor. Table S4 RSCU in the high expression group, low expression group of CD2AP gene. Table S5. RSCU in the high expression group, low expression group of BACH2.

## Abbreviations

The following abbreviations are used in this manuscript:

PAMs	Porcine alveolar macrophages
CUB	Codon usage bias
RSCU	Relative synonymous codon usage
ENC (Nc)	Effective number of codons
CAI	Codon adaptation index
GC3s	GC content at the third codon position
GC12	Mean GC content at the first and second codon positions
PR2	Parity Rule 2
PCA	Principal component analysis
CDS	Coding sequence
ORF	Open reading frame
NJ	Neighbor-joining
TF	Transcription factor
