# Supplementary material for: Comparative Codon Usage Bias of CD2AP and BACH2 Across 49 Vertebrates: Implications for Porcine Macrophage Immunity in Mycoplasma hyopneumoniae Infection
Source: Biology (Basel). 2026 Feb 27;15(5):389. doi: 10.3390/biology15050389 (PMC12984098; doi:10.3390/biology15050389)
Supplement: Supplementary file 1 [file biology-15-00389-s001.zip › supplementary.pdf]

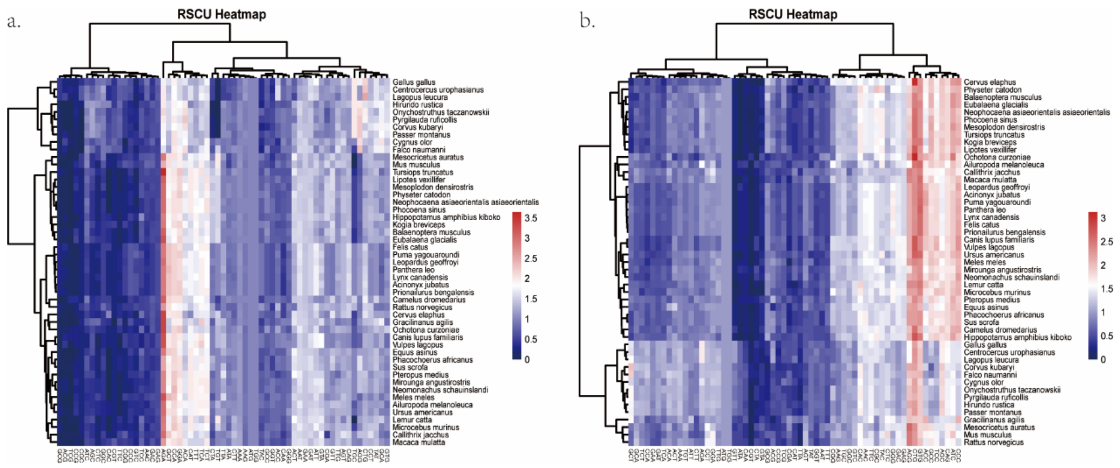

Figure S1. RSCU heatmaps and hierarchical clustering across species.

Heatmaps show the relative synonymous codon usage (RSCU) profiles of (a) CD2AP and (b) BACH2 across 49 species. Rows represent species and columns represent synonymous codons. Colour intensity indicates RSCU values (blue, lower usage; red, higher usage; scale shown at right). Dendrograms depict hierarchical clustering of species (left) and codons (top) based on similarity in their RSCU patterns.

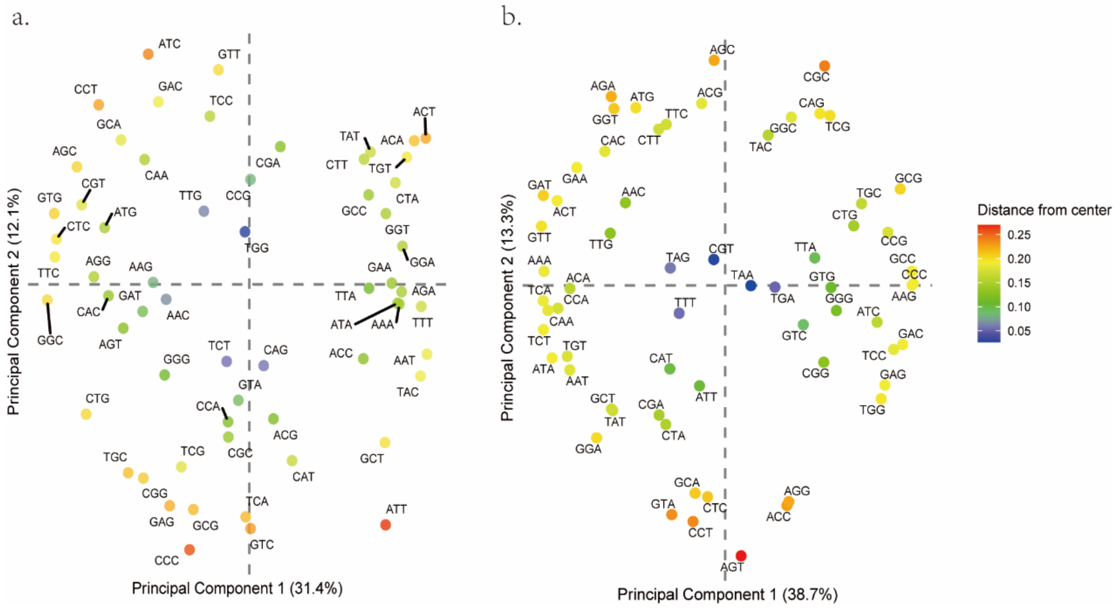

Figure S2. Codon-level PCA loading plots based on RSCU

Codon-level PCA loading plots for (a) CD2AP and (b) BACH2 derived from the RSCU matrix (61 sense codons; stop codons excluded). Each point represents a synonymous codon positioned by its loading on PC1 and PC2. Colours indicate the Euclidean distance from the origin (higher values denote stronger contributions to the corresponding principal components).

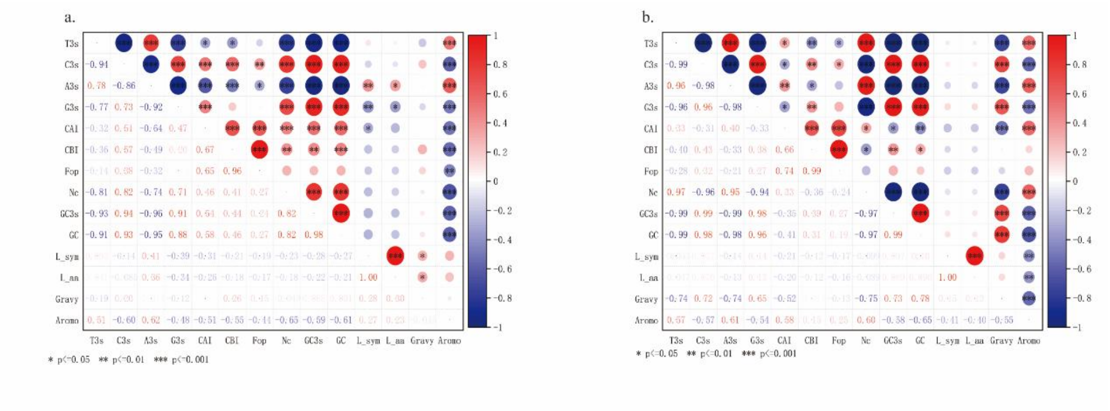

Figure S3. Correlation matrices of codon-usage indices

Correlation matrices for (a) CD2AP and (b) BACH2 across 49 species. Variables include third-position nucleotide frequencies (T3s, C3s, A3s, G3s), codon-bias indices (CAI, CBI, Fop, ENC), GC metrics (GC3s, GC), and protein features (e.g., L_sym, L_aa, Gravy, Aromo). Circle size and colour reflect correlation strength and direction (red, positive; blue, negative). Significance levels are indicated as $P < 0.05$, $P < 0.01$, $P < 0.001$.
